# Glypican-1 Mediates Both Prion Protein Lipid Raft Association and Disease Isoform Formation

**DOI:** 10.1371/journal.ppat.1000666

**Published:** 2009-11-20

**Authors:** David R. Taylor, Isobel J. Whitehouse, Nigel M. Hooper

**Affiliations:** Proteolysis Research Group, Institute of Molecular and Cellular Biology, Faculty of Biological Sciences, and Leeds Institute of Genetics, Health and Therapeutics, University of Leeds, Leeds, United Kingdom; University of Alberta, Canada

## Abstract

In prion diseases, the cellular form of the prion protein, PrP^C^, undergoes a conformational conversion to the infectious isoform, PrP^Sc^. PrP^C^ associates with lipid rafts through its glycosyl-phosphatidylinositol (GPI) anchor and a region in its N-terminal domain which also binds to heparan sulfate proteoglycans (HSPGs). We show that heparin displaces PrP^C^ from rafts and promotes its endocytosis, suggesting that heparin competes with an endogenous raft-resident HSPG for binding to PrP^C^. We then utilised a transmembrane-anchored form of PrP (PrP-TM), which is targeted to rafts solely by its N-terminal domain, to show that both heparin and phosphatidylinositol-specific phospholipase C can inhibit its association with detergent-resistant rafts, implying that a GPI-anchored HSPG targets PrP^C^ to rafts. Depletion of the major neuronal GPI-anchored HSPG, glypican-1, significantly reduced the raft association of PrP-TM and displaced PrP^C^ from rafts, promoting its endocytosis. Glypican-1 and PrP^C^ colocalised on the cell surface and both PrP^C^ and PrP^Sc^ co-immunoprecipitated with glypican-1. Critically, treatment of scrapie-infected N2a cells with glypican-1 siRNA significantly reduced PrP^Sc^ formation. In contrast, depletion of glypican-1 did not alter the inhibitory effect of PrP^C^ on the β-secretase cleavage of the Alzheimer's amyloid precursor protein. These data indicate that glypican-1 is a novel cellular cofactor for prion conversion and we propose that it acts as a scaffold facilitating the interaction of PrP^C^ and PrP^Sc^ in lipid rafts.

## Author Summary

The prion diseases are unique in that their infectious nature is not dependent on nucleic acid but is instead attributed to a misfolded protein, the prion protein. This misfolded prion protein is capable of inducing the misfolding of the normal form of the prion protein that is present on the surface of neurons and other cells in the body. However, the site in the cell at which this misfolding occurs and whether other proteins are involved remains controversial. We have addressed these questions by investigating how the normal form of the prion protein is targeted to specialised domains on the plasma membrane termed cholesterol-rich lipid rafts. We show that targeting is due, in part, to a particular heparin sulfate proteoglycan called glypican-1. Significantly, reducing the levels of glypican-1 in an infected cell line reduced the accumulation of misfolded prion protein. We propose that glypican-1 acts as a scaffold facilitating the favourable interaction of the misfolded, infectious form of the prion protein with the normal cellular form within cholesterol-rich lipid rafts. Our results indicate that glypican-1 is intimately involved in the misfolding of the prion protein, the critical event in the pathogenesis of prion diseases such as Creutzfeldt-Jakob disease in humans.

## Introduction

Creutzfeldt-Jakob (CJD) disease of humans, bovine spongiform encephalopathy of cattle and scrapie of sheep are all examples of prion diseases. These diseases propagate through the misfolding of the normal cellular form of the prion protein (PrP^C^) into the disease-associated isoform (PrP^Sc^) [1]. The conversion of PrP^C^ to PrP^Sc^ is accompanied by a large increase in the β-sheet content of the protein and a propensity to aggregate into larger macromolecular structures. PrP^C^ is post-translationally modified with a glycosyl-phosphatidylinositol (GPI) anchor attached to the C-terminus. The GPI anchor facilitates the association of PrP^C^ with cholesterol- and sphingolipid-rich membrane microdomains, termed lipid rafts (reviewed in [2]). Lipid rafts are characterised biochemically by their resistance to solubilisation with detergents, such as Triton X-100, at low temperature, with the resulting detergent-resistant membranes (DRMs) enriched in raft resident proteins and lipids [3]. PrP^C^ also associates with lipid rafts by virtue of raft targeting determinants within its N-terminal domain [4],[5]. However, the identity of the raft interacting partner(s) for the N-terminal domain of PrP^C^ remains unknown.

A number of studies suggest that the formation of PrP^Sc^ takes place in lipid rafts. For example, PrP^Sc^, like PrP^C^, is present in DRMs isolated from cultured cells [6],[7]. Furthermore, when the GPI anchor of PrP^C^ is replaced by a transmembrane anchor, the protein redistributes to non-raft regions of the plasma membrane and no conversion occurs [7],[8]. In addition, depletion of cellular cholesterol levels to disrupt lipid rafts leads to a reduction in the PrP^Sc^-load in infected cell culture models [8]–[10].

The presence of prion conversion cofactors in a given subcellular location may rationalise why a particular site is favoured for conversion [11]. Of these potential cofactors, there is evidence linking proteoglycans and their glycosaminoglycan (GAG) side chains to PrP^C^ metabolism [12]. Sulfated GAGs, including heparan sulfate, were identified as constituents of PrP^Sc^ plaques in the brains of CJD, Gerstmann-Straussler-Scheinker disease and kuru cases, as well as in hamster brains infected with scrapie [13],[14]. The N-terminal half of PrP^C^ has been shown to bind heparan sulfate [15],[16], with the basic residues at the extreme N-terminus of mature PrP^C^ constituting a particularly strong site of interaction [17]–[19]. GAGs stimulated the endocytosis of chicken PrP^C^ [15] and heparin reduced the level of cell surface human PrP^C^ by an unknown mechanism [17]. Furthermore, the incorporation of PrP^Sc^ into Chinese Hamster Ovary cells required endogenous GAG expression [20] and heparan sulfate acted as a cellular receptor for prion rods in neuroblastoma cells [21]. However, despite all these observations, the identity of the cellular heparan sulfate involved in the interaction with PrP^C^ and/or PrP^Sc^, whether it is lipid raft-associated and involved in the conformational conversion of PrP^C^ to PrP^Sc^ remain to be determined.

In the current study we show that heparin promotes the endocytosis of mammalian PrP^C^ and displaces it from lipid rafts, suggesting that heparin competes with an endogenous raft-resident heparan sulfate proteoglycan (HSPG) for binding to PrP^C^. We then utilised a transmembrane-anchored construct of PrP, PrP-TM [22], which associates with DRMs solely through the raft targeting determinant in its N-terminal domain [5], to identify the GPI-anchored HSPG glypican-1 as a molecule that targets PrP^C^ to detergent-resistant lipid rafts. In addition, we show that glypican-1 directly interacts with both PrP^C^ and PrP^Sc^ and that depletion of glypican-1 in scrapie-infected murine neuroblastoma N2a (ScN2a) cells inhibits the formation of PrP^Sc^.

## Results

### Heparin stimulates the endocytosis of PrP^C^ and displaces it from lipid rafts

**Figure 1:**
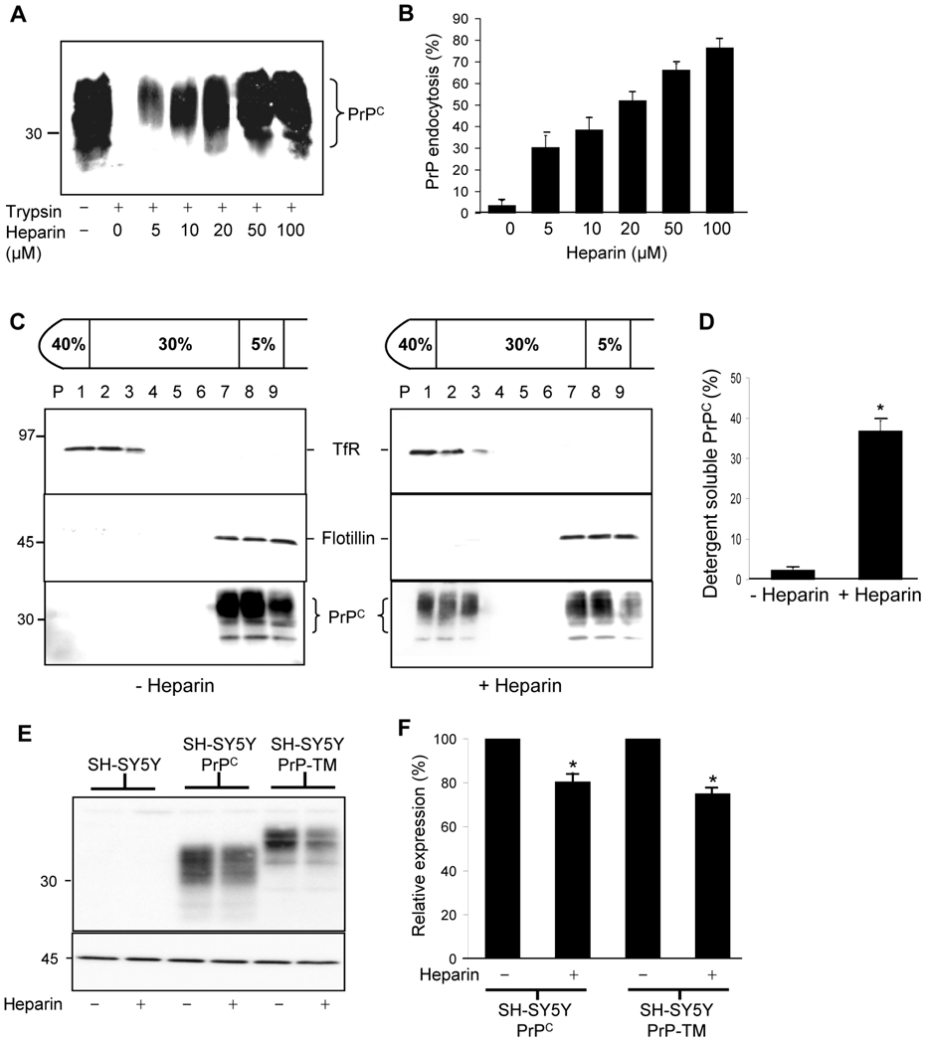
Heparin stimulates the endocytosis of PrP^C^ in a dose-dependent manner and displaces it from detergent-resistant lipid rafts. (**A**) SH-SY5Y cells expressing PrP^C^ were surface biotinylated and then incubated for 1 h at 37°C in the absence or presence of various concentrations of heparin diluted in OptiMEM. Prior to lysis cells were, where indicated, incubated with trypsin to digest cell surface PrP^C^. Cells were then lysed and PrP^C^ immunoprecipitated from the sample using antibody 3F4. Samples were subjected to SDS PAGE and western blot analysis and the biotin-labelled PrP^C^ detected with peroxidase-conjugated streptavidin. (**B**) Densitometric analysis of multiple blots from four separate experiments as described in (A) is shown. (**C**) SH-SY5Y cells expressing PrP^C^ were surface biotinylated and then incubated in the absence or presence of 50 µM heparin prepared in OptiMEM for 1 h at 37°C. Cells were homogenised in the presence of 1% (v/v) Triton X-100 and subjected to buoyant sucrose density gradient centrifugation. PrP^C^ was immunoprecipitated from equal volumes of each gradient fraction using 3F4 and subjected to SDS-PAGE and western blotting. The gradient fractions from both the untreated and heparin treated cells were analysed on the same SDS gel and immunoblotted under identical conditions. The biotin-labelled PrP^C^ was detected with peroxidase-conjugated streptavidin. Flotillin-1 and transferrin receptor (TfR) were detected by immunoblotting as markers for DRM and detergent-soluble fractions, respectively. (**D**) Densitometric analysis of the proportion of total PrP^C^ in the detergent soluble fractions of the plasma membrane. (**E**) Untransfected SH-SY5Y cells and SH-SY5Y cells expressing either PrP^C^ or PrP-TM were grown to confluence and then incubated for 1 h in the presence or absence of 50 µM heparin prepared in OptiMEM. Media samples were collected and concentrated and cells harvested and lysed. Cell lysate samples were immunoblotted for PrP^C^ using antibody 3F4, with β-actin used as a loading control. (**F**) Quantification of PrP^C^ and PrP-TM levels after treatment of cells with heparin as in (E). Experiments were performed in triplicate and repeated on three occasions. * P<0.05.

To investigate whether heparin promotes the endocytosis of mammalian PrP^C^, and to determine the mechanism involved, we utilised human neuroblastoma SH-SY5Y cells stably expressing murine PrP^C^ (tagged with the 3F4 antibody epitope) and an established endocytosis assay [23],[24]. It should be noted that the SH-SY5Y cells do not express detectable levels of endogenous PrP^C^ (see Fig. 1E) as reported previously [19],[25],[26]. In the endocytosis assay, plasma membrane proteins are selectively labelled using a cell-impermeable biotin reagent, thus allowing their distinction from proteins either in the secretory pathway or already endocytosed. Cells were then incubated with heparin for 1 h at 37°C and subsequently treated with trypsin to remove residual cell surface PrP^C^ prior to lysis. Any PrP^C^ endocytosed during the course of the experiment is protected from trypsin digestion. In the absence of heparin, negligible (4%) cell surface PrP^C^ was endocytosed, whereas heparin stimulated the endocytosis of PrP^C^ in a dose-dependent manner, with 77% internalised by 100 µM heparin in 1 h (Fig. 1A and B).

Cu^2+^ ions stimulate the clathrin-dependent endocytosis of PrP^C^ by a mechanism which is initiated by the protein dissociating from lipid rafts [24]. Therefore, we determined whether heparin could likewise displace PrP^C^ from lipid rafts. Cells were first surface biotinylated and then incubated with heparin before homogenisation in the presence of Triton X-100 followed by buoyant sucrose density gradient centrifugation. Consistent with previous reports [5],[24], PrP^C^ in cells incubated in the absence of heparin resided almost exclusively at the 5%/30% sucrose interface in the DRM fractions containing the raft-associated protein, flotillin-1 (Fig. 1C). However, in cells incubated with heparin, a significant amount (37%) of PrP^C^ redistributed to detergent-soluble fractions of the plasma membrane isolated at the bottom of the sucrose gradient containing the transferrin receptor (TfR, Fig. 1C and D). To determine whether the heparin treatment had any effect on other aspects of PrP^C^ metabolism, we investigated the effect of the 1 h heparin treatment on the expression and shedding of PrP^C^ in untransfected and PrP^C^-transfected SH-SY5Y cells (Fig. 1E). Endogenous PrP^C^ expression in untransfected SH-SY5Y cells remained undetectable following heparin treatment (Fig. 1E). In the SH-SY5Y cells transfected with PrP^C^, 1 h heparin treatment led to 19.5% reduction in the total cellular amount of PrP^C^ (Fig. 1E and F) which may be attributable to its degradation following endocytosis. No PrP^C^ was detected in concentrated conditioned media in the presence or absence of heparin treatment (data not shown). Together, these data indicate that the addition of heparin to cells for 1 h does not increase the expression or shedding of PrP^C^ but stimulates the endocytosis of mammalian PrP^C^ by its lateral displacement from detergent-resistant lipid rafts into detergent-soluble regions of the plasma membrane. From this, we hypothesised that the lateral movement of PrP^C^ from rafts may occur as exogenous heparin competes with an endogenous raft-resident HSPG for binding to PrP^C^.

### PrP-TM interacts with DRMs through association with the GPI-anchored HSPG glypican-1

**Figure 2:**
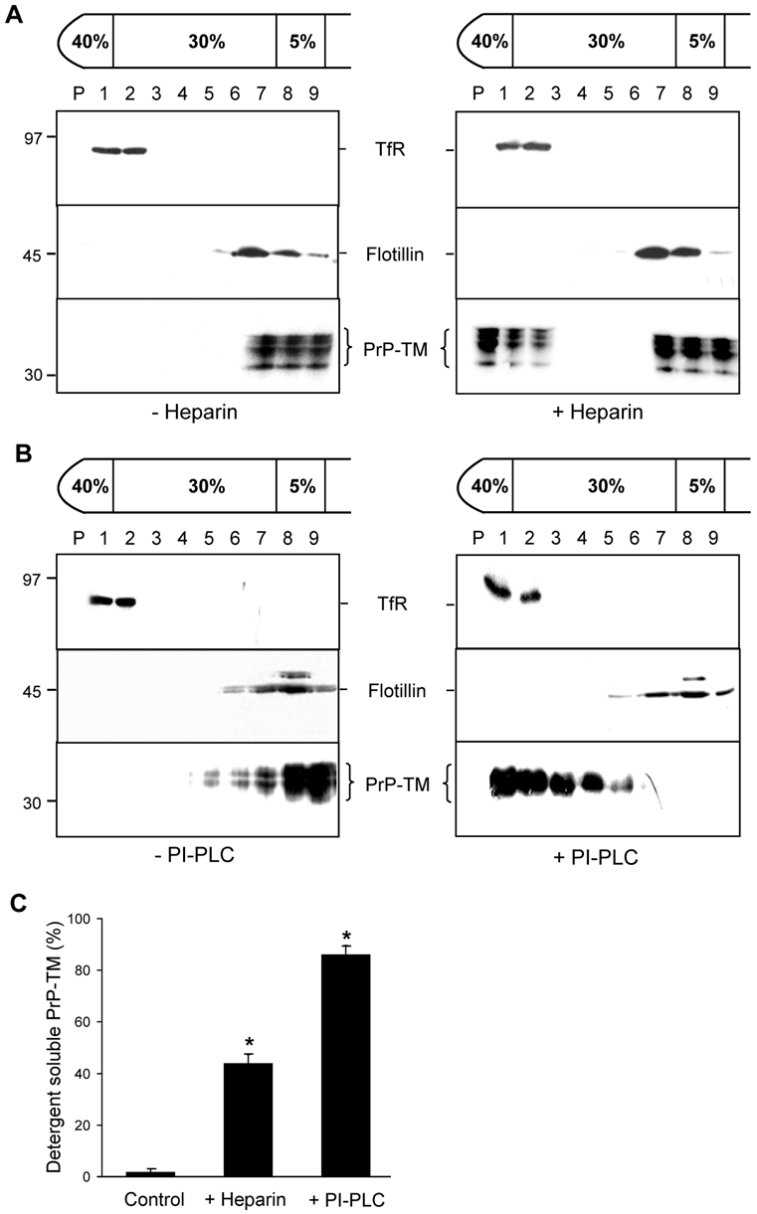
The association of PrP-TM with DRMs is disrupted by treatment of cells with either heparin or bacterial PI-PLC. SH-SY5Y cells expressing PrP-TM were surface biotinylated and then (**A**) incubated in the absence or presence of 50 µM heparin prepared in OptiMEM for 1 h at 37°C or (**B**) incubated in the absence or presence of 1 U/ml bacterial PI-PLC for 1 h at 4°C. Cells were homogenised in the presence of 1% (v/v) Triton X-100 and subjected to buoyant sucrose density gradient centrifugation. PrP-TM was immunoprecipitated from equal volumes of each gradient fraction using 3F4 and subjected to western blotting. The biotin-labelled PrP-TM fraction was detected with peroxidase-conjugated streptavidin. Flotillin-1 and transferrin receptor (TfR) were detected by immunoblotting as markers for DRM and detergent-soluble fractions respectively. (**C**) Densitometric analysis of the proportion of total PrP-TM present in the detergent soluble fractions of the plasma membrane after heparin and PI-PLC treatment. Experiments were performed in triplicate and repeated on three occasions. * P<0.05.

To test this hypothesis we employed a transmembrane-anchored construct of PrP (PrP-TM) in which the GPI anchor signal sequence of murine PrP is replaced with the transmembrane and cytosolic domains of the non-raft protein angiotensin converting enzyme [22]. PrP-TM also contains the 3F4 epitope. While wild-type PrP^C^ is targeted to DRMs by virtue of both its GPI anchor and lipid raft targeting determinants in its N-terminal domain, PrP-TM localises to DRMs solely by means of its N-terminal domain interacting with raft resident molecule(s) [5]. Thus, PrP-TM offers a unique system to identify cellular components that interact with the N-terminal domain of PrP^C^ and target it to lipid rafts. SH-SY5Y cells expressing PrP-TM were surface biotinylated and then incubated with heparin prior to DRM isolation. As reported previously [5],[24], PrP-TM resided exclusively in the DRM fractions of the plasma membrane (Fig. 2A). However, in those cells that had been incubated with heparin, 44% of PrP-TM was now localised to the detergent-soluble fractions at the base of the sucrose gradient (Fig. 2A and C). As with wild type PrP^C^, the heparin treatment did not increase the expression or stability of PrP-TM (Fig. 1E and F). In parallel, SH-SY5Y cells expressing PrP-TM were incubated with bacterial phosphatidylinositol-specific phospholipase C (PI-PLC) to determine whether the lipid raft targeting of PrP-TM was due to raft-resident GPI-anchored proteins (Fig. 2B). Treatment of cells with PI-PLC after cell surface biotinylation almost completely abrogated PrP-TM association with the DRMs but had no effect on the DRM distribution of flotillin-1 (Fig. 2B and C), indicating that gross disruption of the rafts had not occurred. One interpretation of the ability of both exogenous heparin and PI-PLC treatment to inhibit the association of PrP-TM with DRMs is that a GPI-anchored HSPG is responsible for the lipid raft targeting of PrP-TM.

**Figure 3:**
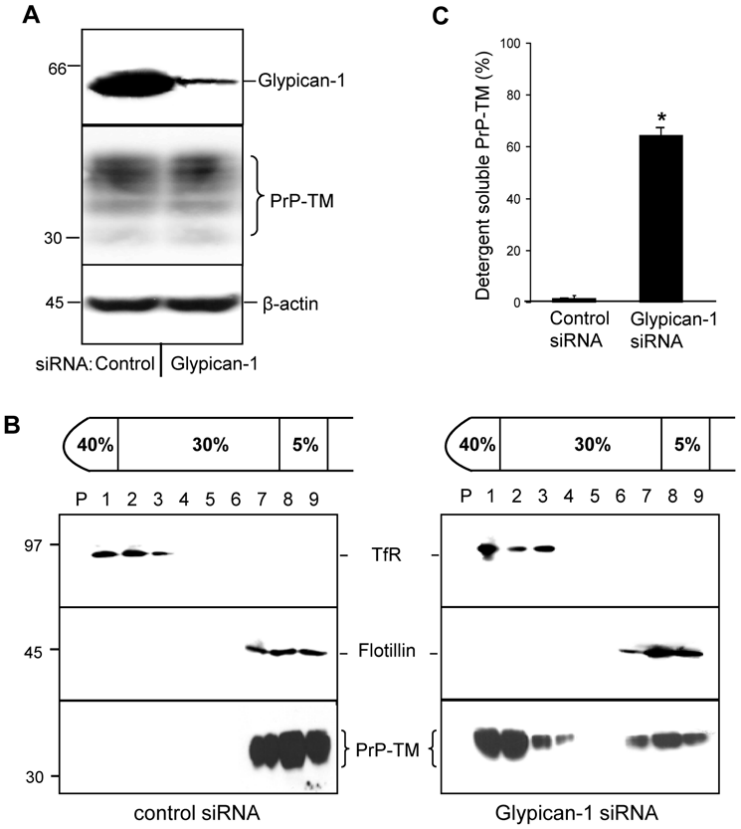
Depletion of glypican-1 inhibits the association of PrP-TM with DRMs. SH-SY5Y cells expressing PrP-TM were treated with either control siRNA or siRNA targeted to glypican-1 and then allowed to reach confluence for 48 h. Cells were subsequently surface biotinylated and incubated in OptiMEM for 1 h at 37°C in the presence of Tyrphostin A23 to block endocytosis. The media was removed and the cells washed in phosphate-buffered saline prior to homogenisation in the presence of 1% (v/v) Triton X-100 and subjected to buoyant sucrose density gradient centrifugation. (**A**) Quantification of glypican-1 and PrP-TM expression in cell lysates. To detect glypican-1, cell lysate samples were treated with heparinase I and heparinase III prior to electrophoresis as described in the materials and methods section. (**B**) PrP-TM was immunoprecipitated from equal volumes of each gradient fraction using 3F4 and then subjected to western blotting with peroxidase-conjugated streptavidin. Flotillin-1 and transferrin receptor (TfR) were detected by immunoblotting as markers for DRM and detergent-soluble fractions, respectively. (**C**) Densitometric analysis of the proportion of total PrP-TM present in the detergent soluble fractions of the plasma membrane after siRNA treatment from multiple blots from three independent experiments. * P<0.05.

The glypicans are a family of GPI-anchored HSPGs [27], of which glypican-1 is particularly abundant in neurons [28]. To assess whether glypican-1 is involved in the interaction of PrP-TM with DRMs, SH-SY5Y cells expressing PrP-TM were transfected with either a control siRNA or a siRNA pool directed against glypican-1. The specific siRNAs reduced the amount of glypican-1 in the cells by 84% (Fig. 3A). The siRNA treated cells were surface biotinylated prior to DRM isolation. In glypican-1 siRNA treated cells a large proportion (64%) of PrP-TM was displaced from the DRMs, instead localising with the detergent-soluble fractions (Fig. 3B and C), indicating that glypican-1 plays a role in the lipid raft targeting of PrP-TM.

### Glypican-1 retains PrP^C^ in lipid rafts at the cell surface

**Figure 4:**
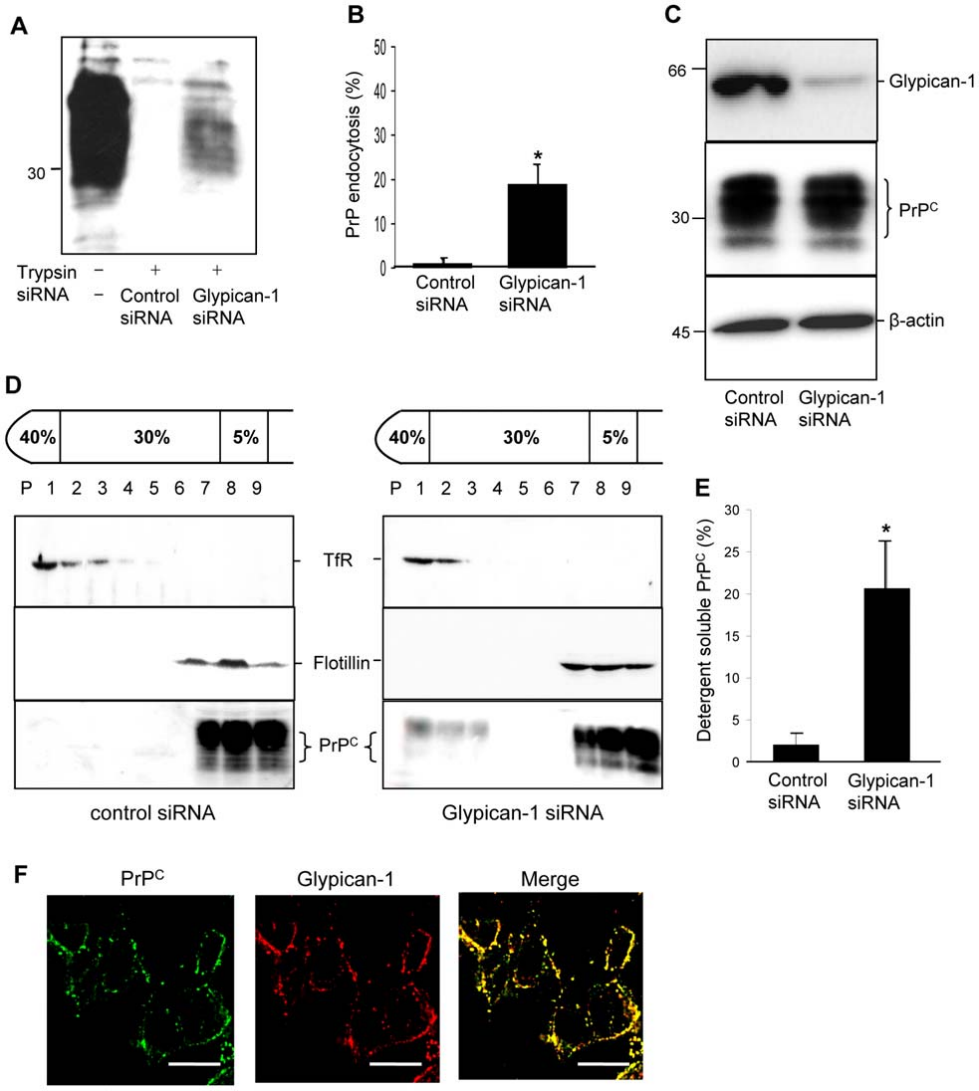
Depletion of glypican-1 stimulates the endocytosis of PrP^C^. SH-SY5Y cells expressing wild type PrP^C^ were treated with either control or glypican-1 siRNA and then incubated for 60 h. Cells were surface biotinylated and incubated in OptiMEM for 1 h at 37°C. Where indicated, cells were treated with trypsin to remove remaining cell surface PrP^C^. Cells were then lysed and total PrP^C^ immunoprecipitated from the sample using antibody 3F4. (**A**) Samples were subjected to western blot analysis and the biotin-labelled PrP^C^ fraction was detected with peroxidase-conjugated streptavidin. (**B**) Densitometric analysis (mean ± s.e.m.) of multiple blots from three separate experiments in (A) is shown. (**C**) Expression of glypican-1 (in lysate samples treated with heparinase I and heparinase III) and PrP^C^ in the cell lysates from (A). β-actin was used as a loading control. (**D**) SH-SY5Y cells expressing PrP^C^ were treated with either control siRNA or glypican-1 siRNA and then allowed to reach confluence for 48 h. Cells were subsequently surface biotinylated and incubated in OptiMEM for 1 h at 37°C. Cells were homogenised in the presence of 1% (v/v) Triton X-100 and subjected to buoyant sucrose density gradient centrifugation. (**E**) Densitometric analysis of the proportion of total PrP^C^ present in the detergent soluble fractions of the plasma membrane after siRNA treatment from three independent experiments. (**F**) SH-SY5Y cells expressing PrP^C^ were seeded onto glass coverslips and grown to 50% confluency. Cells were fixed, and then incubated with anti-PrP antibody 3F4 and a glypican-1 polyclonal antibody. Finally, cells were incubated with Alexa488-conjugated rabbit anti-mouse and Alexa594-conjugated goat anti-rabbit antibodies and viewed using a DeltaVision Optical Restoration Microscopy System. Images are representative of three individual experiments. Scale bars equal 10 µm. * P<0.05.

Next, we sought to determine if glypican-1 was involved in the lipid raft targeting of wild type PrP^C^. We reasoned that if glypican-1 is involved in the raft targeting of PrP^C^, then its depletion should increase the endocytosis of PrP^C^. SH-SY5Y cells expressing PrP^C^ were treated with either control siRNA or siRNA against glypican-1. Cells were then surface biotinylated, incubated for 1 h at 37°C and the amount of PrP^C^ endocytosed determined. In those cells treated with the control siRNA, incubation in serum-free medium resulted in little detectable endocytosis of PrP^C^ (Fig. 4A and B). However, in the cells treated with the glypican-1 siRNA, a modest but significant amount (19%) of PrP^C^ was endocytosed (Fig. 4A and B). In the cells treated with the glypican-1 siRNA, a significant knockdown (88%) of glypican-1 was clearly apparent, with no detectable effect on the amount of PrP^C^ (Fig. 4C).

We also assessed the raft distribution of PrP^C^ in cells treated with control or glypican-1 siRNA. Cells were then surface biotinylated and incubated for 1 h at 37°C in the presence of tyrphostin A23, to block clathrin-mediated endocytosis [24]. In control siRNA treated cells, cell surface PrP^C^ resided exclusively in the DRMs (Fig. 4D). In contrast, in cells treated with glypican-1 siRNA a significant amount (20.6%) of cell surface PrP^C^ localised to the detergent-soluble fractions of the sucrose gradient (Fig. 4D and E). Immunofluorescence microscopy revealed that PrP^C^ and glypican-1 extensively colocalised on the surface of the cells (Fig. 4F). Collectively these data indicate that PrP^C^ and glypican-1 co-localise on the cell surface and that knockdown of glypican-1 allows PrP^C^ to translocate out of the detergent-insoluble rafts into detergent-soluble regions of the plasma membrane prior to its endocytosis.

### PrP^C^ and PrP^Sc^ interact with glypican-1

**Figure 5:**
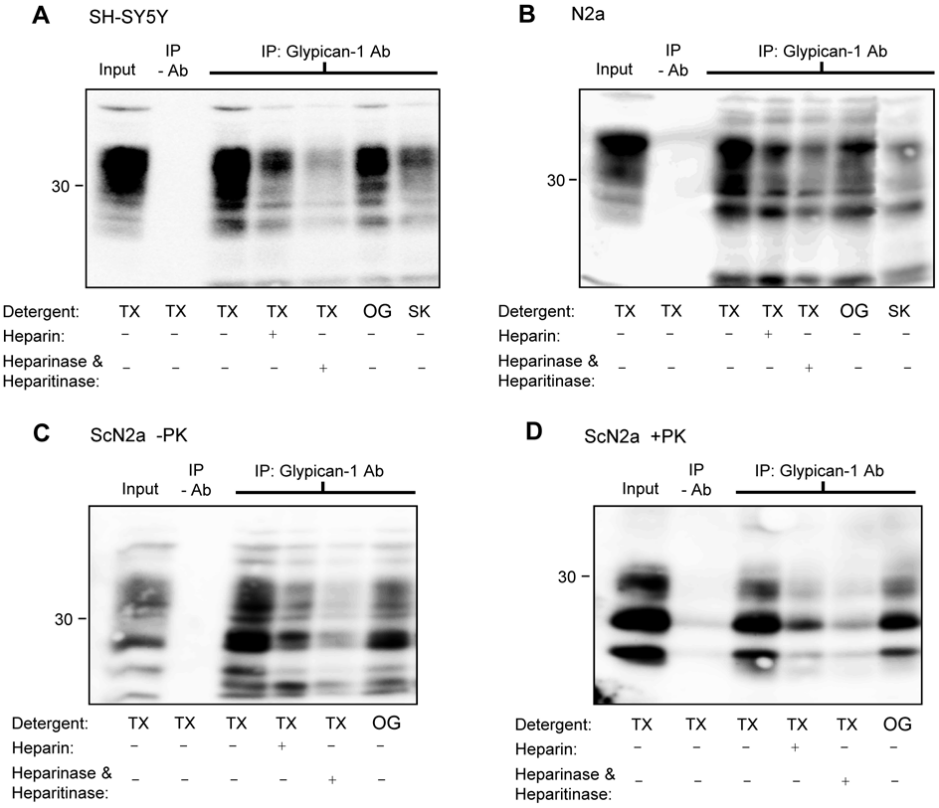
PrP^C^ and PrP^Sc^ immunoprecipitate with glypican-1. (**A**) SH-SY5Y cells expressing PrP^C^, (**B**) N2a cells or (**C and D**) ScN2a cells were lysed in the indicated detergents and then immunoprecipitated with a polyclonal glypican-1 antibody and where indicated, co-incubated with 50 µM heparin. Those samples pretreated with heparinase I and heparinase III were lysed with Triton X-100 followed by immunoprecipitation with glypican-1 antibody. In (D), immunopreciptiates from ScN2a cells were digested with PK. All immunoprecipitates were subsequently blotted for PrP. TX, Triton X-100; OG, octylglucoside; SK, sarkosyl.

We next sought to determine if glypican-1 is able to interact directly with PrP^C^ and PrP^Sc^ by co-immunoprecipitation. When cell lysates prepared using Triton X-100 from SH-SY5Y cells stably expressing PrP^C^ (Fig. 5A) or N2a cells endogenously expressing PrP^C^ (Fig. 5B) were incubated with a polyclonal antibody against glypican-1, PrP^C^ was co-immunoprecipitated in both cases (Fig. 5A). The interaction between glypican-1 and PrP^C^ was disrupted by prior treatment of the cell lysates with heparinase I and heparinase III to digest the heparan sulfate chains on glypican-1 [29],[30] or exogenous heparin (Fig. 5A and B), indicating that the interaction between PrP^C^ and glypican-1 involves the heparan sulfate sidechains on the latter. In order to provide further evidence that glypican-1 and PrP^C^ are directly associated, co-immunoprecipitation experiments were performed using cells lysed with sarkosyl or octylglucoside, detergents known to completely solubilise lipid rafts [31]–[33]. PrP^C^ co-immunoprecipitated with glypican-1 using lysates prepared with either detergent (Fig. 5A and B).

To address whether glypican-1 was also able to interact with PrP^Sc^ we performed co-immunoprecipitation experiments using cell extracts prepared from mouse neuroblastoma cells persistently infected with PrP^Sc^ (ScN2a cells). Immunoprecipitation of glypican-1 led to the co-immunoprecipitation of PrP from cells lysed with either Triton X-100 or octylglucoside (Fig. 5C). Sarkosyl is unsuitable for use in co-immunoprecipitation experiments in ScN2a cells owing to its ability to aggregate PrP^Sc^ [34]. Co-immunoprecipitation of PrP with glypican-1 in Triton-extracted cells was inhibited by co-incubation with exogenous heparin or removal of the heparan sulfate sidechains on glypican-1 with heparinase I and heparinase III treatment (Fig. 5C). When the protein A-sepharose pellets were treated with proteinase K (PK) prior to subsequent analysis, PrP^Sc^ was found to have precipitated with glypican-1 in both the Triton X-100 and octylglucoside extracted cells (Fig. 5D). Similarly, the interaction between glypican-1 and PrP^Sc^ was inhibited by addition of exogenous heparin to the cell lysates or pre-treatment of samples with heparinase I and heparinase III (Fig. 5D). These data indicate that glypican-1 is capable of interacting via its heparan sulfate chains with both normal and protease-resistant forms of PrP.

### Depletion of glypican-1 inhibits the formation of PrP^Sc^

**Figure 6:**
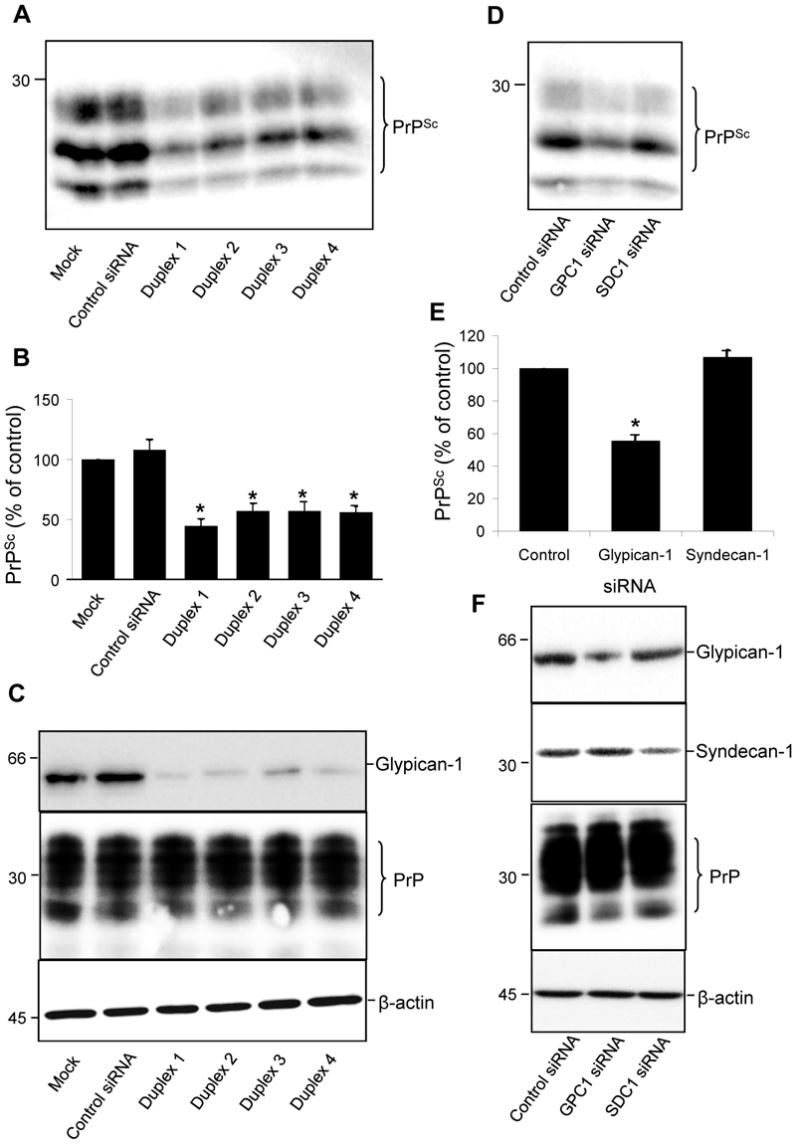
Depletion of glypican-1 by siRNA reduces PrP^Sc^ formation. ScN2a cells were either untreated or incubated with either control siRNA or one of four siRNAs targeted to glypican-1. After 48 h incubation the treatments were repeated. After a total incubation period of 96 h cells were harvested, lysed and protein concentration determined. (**A**) For detection of PK-resistant PrP^Sc^, samples containing 200 µg protein were digested with 4 µg PK for 30 min at 37°C. Protein was then recovered by methanol precipitation and immunoblotted for PrP using antibody 6D11. (**B**) Densitometric analysis (mean ± s.e.m.) of PK-resistant PrP^Sc^ levels for each treatment, relative to those of mock-treated cells, from multiple blots from four independent experiments. (**C**) To confirm that glypican-1 depletion had been achieved in the ScN2a cells, cell lysate samples were immunoblotted for glypican-1, as well as PrP and β-actin. (**D**) To confirm the specificity of glypican-1 in modulating PrP^Sc^ formation, ScN2a cells were treated with control, syndecan-1 or glypican-1 siRNA reagents. Samples were processed as described in (A). (**E**) Densitometric analysis (mean ± s.e.m.) of PK-resistant PrP^Sc^ levels for each treatment in (D), from multiple blots from three independent experiments. (**F**) Confirmation of syndecan-1 and glypican-1 knockdown by immunblotting.

Next we investigated if the interaction with glypican-1 could modulate the conversion of PrP^C^ to PrP^Sc^. ScN2a cells were treated with the control siRNA or one of four siRNA duplexes targeted to mouse glypican-1. After a total incubation period of 96 h, cells were harvested, lysed, and then the cell lysates digested with PK prior to SDS-PAGE and subsequent immunoblotting (Fig. 6A). Treatment with the control siRNA did not alter the level of PK-resistant PrP^Sc^. However, in cells treated with each of the four glypican-1 siRNA duplexes an average 50% reduction in PK-resistant PrP^Sc^ was consistently observed (Fig. 6A and B). Knockdown (78–87%) of glypican-1 by the siRNA duplexes in the ScN2a cells was confirmed by immunoblotting (Fig. 6C). The reduction in PK-resistant PrP^Sc^ seen in the glypican-1 depleted cells was not a consequence of a more general reduction in PrP levels, as when non-PK digested samples were immunoblotted the amount of total PrP was unaltered relative to the control siRNA treated cells (Fig. 6C).

Next we sought to confirm the specificity of glypican-1 in modulating the conversion of PrP^C^ to PrP^Sc^ by testing whether another HSPG, syndecan-1, could influence PrP^Sc^ accumulation in ScN2a cells. When cells were treated with syndecan-1 siRNA over an incubation period of 96 h, the level of PK-resistant PrP^Sc^ remained comparable to control siRNA treated cells (Fig. 6D and E). In contrast, treatment of ScN2a cells with glypican-1 siRNA once more led to 50% reduction in PK-resistant PrP^Sc^ (Fig. 6D and E). Knockdown of glypican-1 (73.3±5.3%) and syndecan-1 (60.7±6.3%) was confirmed by immunoblotting (Fig. 6F).

**Figure 7:**
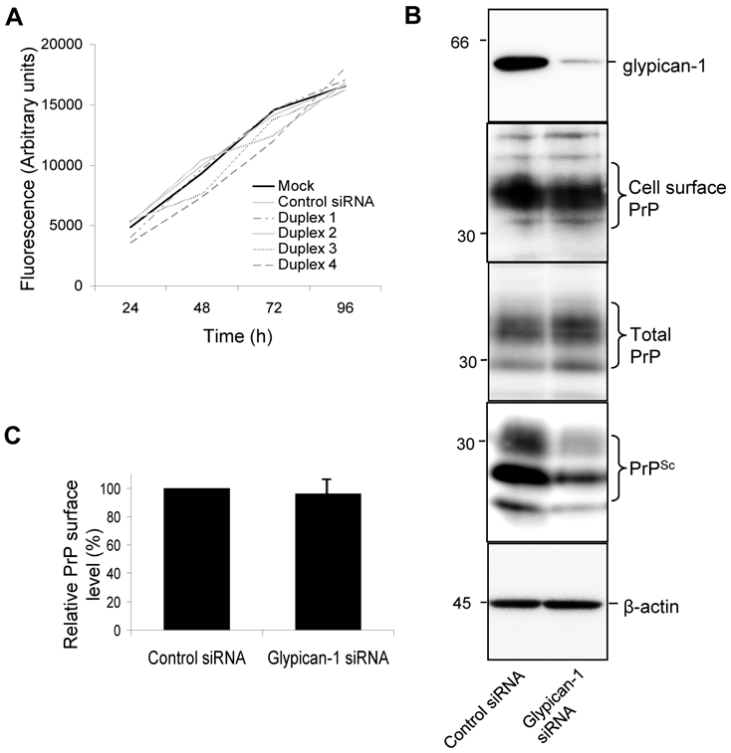
Depletion of glypican-1 does not affect cell division or surface levels of PrP^C^. (**A**) ScN2a cells were seeded into 96 well plates and treated with transfection reagent only or incubated with either control siRNA or one of the four siRNAs targeted to glypican-1. Those experiments exceeding 48 h were dosed with a second treatment of the indicated siRNAs. Cells were then rinsed with PBS and fixed with 70% (v/v) ethanol. Plates were allowed to dry, stained with Hoescht 33342 and the fluorescence measured. (**B**) ScN2a cells were treated with control or glypican-1 siRNA. After 96 h, cell monolayers were labelled with a membrane impermeable biotin reagent. Biotin-labelled cell surface PrP was detected by immunoprecipitation using 6D11 and subsequent immunoblotting using HRP-conjugated streptavidin. Total PrP and PK-resistant PrP (PrP^Sc^) were detected by immunoblotting using antibody 6D11. (**C**) Densitometric analysis of the proportion of the relative amount of biotinylated cell surface PrP in the absence or presence of glypican-1 siRNA from three independent experiments.

Cell division can modulate the accumulation of prions in ScN2a cells [35] and glypican-1 depletion has been reported to inhibit cell proliferation in endothelial cells by arresting cell cycle progression [36]. Therefore, we investigated whether the effect of glypican-1 knockdown was altering the amount of PrP^Sc^ in the ScN2a cells due to modulation of cell division. However, glypican-1 depletion did not significantly affect cell proliferation compared to mock-treated and control siRNA-treated cells (Fig. 7A). As the cell surface is the probable site for the initial interaction between the two isoforms of PrP [11], we assessed whether the reduction in PrP^Sc^ associated with glypican-1 depletion was due to an alteration in the steady state level of PrP at the cell surface. ScN2a cells were surface biotinylated following treatment with either control siRNA or glypican-1 siRNA. Although there was a reduction in the amount of PrP^Sc^ in the glypican-1 siRNA treated cells, there was no difference in the amount of biotinylated cell surface PrP (Fig. 7B and C).

### Glypican-1 is not involved in the inhibitory effect of PrP^C^ on the BACE1 cleavage of APP

**Figure 8:**
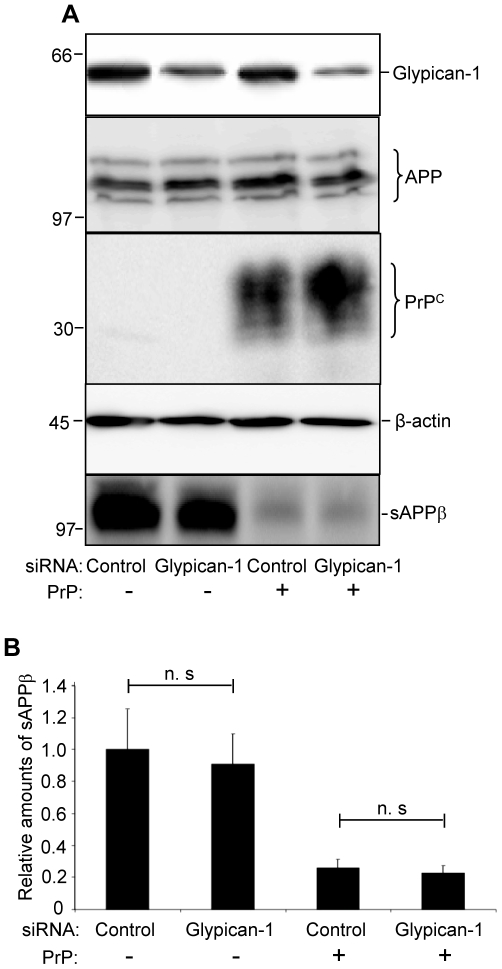
Depletion of glypican-1 does not affect the inhibition of BACE1 by PrP^C^. SH-SY5Y cells co-expressing APP695 and PrP or cells expressing APP695 and an empty pIRES*neo* vector were treated with control siRNA or siRNA targeted to glypican-1. After 30 h the medium was replaced and cells incubated with reduced-serum medium for a further 24 h. Conditioned medium was harvested and cell lysates prepared. (**A**) Expression of glypican-1, full-length APP and PrP^C^ in cell lysates, with β-actin as a loading control. Immunodetection of sAPPβ in conditioned medium. (**B**) Densitometric analysis of sAPPβ levels in glypican-1 depleted cells relative to control siRNA cells, calculated from multiple blots from three independent experiments.

We have recently reported that PrP^C^ inhibits the cleavage of the amyloid precursor protein (APP) by the β-secretase BACE1 [19]. This effect was dependent on the lipid raft localisation of PrP^C^ and was mediated by the N-terminal polybasic region of PrP^C^ through interaction with GAGs [19]. Therefore, we hypothesised that glypican-1 may be involved in the mechanism by which PrP^C^ regulates the BACE1 cleavage of APP. To address this, control siRNA or siRNA directed against glypican-1 were transfected into SH-SY5Y cells expressing PrP^C^. Parallel experiments were performed in SH-SY5Y cells transfected with an empty pIRES*neo* vector. In the media from control siRNA-treated cells expressing PrP^C^ the level of sAPPβ, the product of BACE1 cleavage of APP, was dramatically reduced relative to cells lacking PrP^C^ (Fig. 8A and B), as seen previously [19]. In media from the empty vector cells treated with glypican-1 siRNA there was no alteration in the level of sAPPβ relative to control siRNA-treated cells (Fig. 8A and B). Significantly, levels of sAPPβ in the cells expressing PrP that had been treated with the glypican-1 siRNA were not altered relative to the control siRNA treated cells (Fig. 8A and B), implying that the GAG sidechains of glypican-1 are not critical in mediating the inhibition of BACE1 by PrP^C^.

## Discussion

By utilising a transmembrane anchored form of PrP, PrP-TM, which associates with DRMs solely by means of its N-terminal domain and not also via the GPI anchor as in wild type PrP^C^, we have identified the GPI-anchored HSPG, glypican-1, as involved in targeting PrP^C^ to detergent-resistant lipid rafts in the plasma membrane of neuronal cells. Furthermore, we have shown that depletion of glypican-1 by siRNA knock down significantly inhibits the formation of PrP^Sc^ in a scrapie-infected cell line. Thus, we have identified glypican-1 as a novel cofactor involved in the cellular conversion of PrP^C^ to PrP^Sc^. Glypican-1 appeared to be interacting via its heparan sulfate chains with both PrP^C^ and PrP^Sc^, as the interaction was inhibited by digestion of its GAG chains with heparinase I and heparinase III. This would be consistent with a previous study, where treatment of ScN2a cells with heparinase III caused a profound reduction in the level of PrP^Sc^ [37].

**Figure 9:**
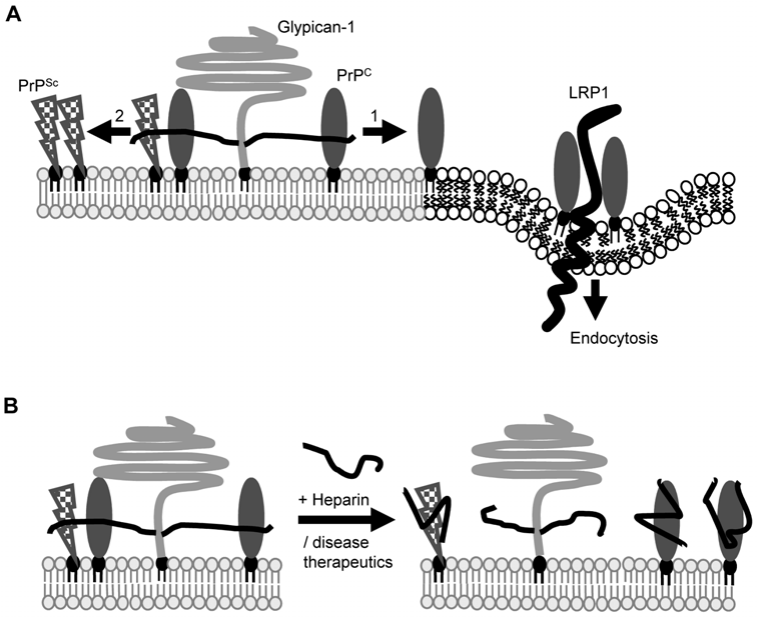
Proposed model for glypican-1 in prion conversion. Under normal circumstances, glypican-1 (light grey) with its heparan sulfate side chains (black lines) constitutes a lipid raft targeting determinant for PrP^C^. (**A**) For PrP^C^ (dark grey) to exit lipid rafts and undergo endocytosis through its interaction with low density lipoprotein receptor-related protein 1 (LRP1, thick black line [59]), its interaction with glypican-1 must be disrupted (arrow 1). In prion disease, we hypothesise that glypican-1 provides a scaffold to facilitate interaction between PrP^C^ and PrP^Sc^ (chequered) and allow misfolding of PrP^C^ to proceed (arrow 2). (**B**) Prion therapeutic strategies may act, in part, by disrupting the interaction of PrP^C^ and PrP^Sc^ that is facilitated through the heparan sulfate sidechains of glypican-1.

We propose a model where glypican-1 acts as a scaffold for prion propagation by binding to both PrP^C^ and PrP^Sc^, thereby bringing them into close enough proximity within lipid rafts to facilitate prion conversion (Fig. 9A). This is supported by the observation that both PrP^C^ and PrP^Sc^ co-immunoprecipitated with glypican-1 and that both isoforms of PrP are localised in lipid rafts [6],[7]. Glypican-1 may be acting as a catalyst, increasing the rate of conversion of PrP^C^ to PrP^Sc^. In this respect, a previous study has demonstrated that pentosan polysulfate destabilised protease-sensitive PrP with a loss of α-helical structure [38]. Thus, a potential mechanism by which glypican-1 may be acting is through its heparan sulfate side chains exerting a similar destabilising effect on the structural stability of PrP^C^, thereby facilitating its refolding in the presence of PrP^Sc^. Although depletion of glypican-1 did not alter the cell surface level of PrP, we cannot exclude the possibility that glypican-1 may also have an effect on the conversion of PrP^C^ to PrP^Sc^ by altering the trafficking or clearance of PrP^Sc^.

Glypican-1 itself is internalised from the plasma membrane by a mechanism involving caveolin-1 [39]. During its intracellular trafficking the heparin sulfate chains of glypican-1 are removed and degraded, either by heparanase cleavage or a non-enzymatic deaminative cleavage which is nitric oxide-catalysed and Cu^2+^-/Zn^2+^-dependent [40]. The nitric oxide is derived from nitrosylated cysteine residues within the glypican-1 core protein, formed via a Cu^2+^-dependent redox reaction [41]. In both cell-free experiments and cell models, Cu^2+^-loaded PrP^C^ was shown to support the nitrosylation of glypican-1 [40]. Recently, it was proposed that glypican-1 autoprocessing is involved in the cellular clearance of PrP^Sc^ [42]. These authors used scrapie-infected hypothalamic (ScGT1) cells and immunofluorescence microscopy to show that PrP^Sc^-associated immunofluorescence was increased in cells treated with a glypican-1 specific siRNA, though no direct quantification was provided [42]. In addition, when ScGT1 cells were treated with reagents to inhibit glypican-1 autoprocessing, western blot analysis revealed increased levels of PrP^Sc^ [42]. From this, these authors argued that glypican-1 autoprocessing may contribute to the cellular clearance of PrP^Sc^, thus inhibiting this process would lead to an increase in PrP^Sc^ accumulation [42]. However, prevention of the autoprocessing of glypican-1 may increase the half-life of the protein, and thus lead to the observed rise in PrP^Sc^ if glypican-1 is a cofactor in prion conversion as our data indicate.

Interestingly, PrP^Sc^ still propagated following the substantial knock down of glypican-1 by siRNA; PrP^Sc^ levels were reduced by at most 55% despite glypican-1 protein levels being reduced by over 80%. One possibility is that the remaining glypican-1 is sufficient to support prion conversion. Alternatively, these data may be interpreted with respect to the protein-only hypothesis in that PrP^Sc^ is sufficient on its own to convert PrP^C^, with glypican-1 acting merely as a catalyst [1]. Another possibility is the existence of other conversion-favouring cellular cofactors, which may include other HSPGs although the lack of effect of syndecan-1 depletion on PrP^Sc^ formation argues against the involvement of this family of proteoglycans. In this context it is also interesting to note that siRNA knock down of glypican-1 did not completely abolish the association of PrP-TM with DRMs, suggesting the existence of additional lipid raft targeting molecules. Such components may be proteins, such as other members of the glypican family [27] or lipids. In support of the latter, PrP lacking its GPI anchor was shown to interact with cholesterol and sphingomyelin containing raft-like lipid vesicles [4]. Whether the same additional molecules involved in the raft targeting of PrP^C^ are also involved in facilitating the conversion of PrP^C^ to PrP^Sc^ awaits their identification and subsequent determination of their role in these processes.

To our knowledge the only other protein to date shown to be capable of influencing prion accumulation in cultured cells is the 37 kDa/67 kDa laminin receptor [43]. When infected cells were depleted of the 37 kDa/67 kDa laminin receptor, PrP^Sc^ levels were significantly reduced, although this may be the result of reduced PrP^C^ expression seen after these treatments rather than the loss of a direct role for the 37 kDa/67 kDa laminin receptor in prion conversion [43]. Interestingly, PrP^C^ interacted with the 37 kDa/67 kDa laminin receptor via both a direct interaction involving residues 144–179 and an indirect interaction via an unidentified HSPG involving residues 53–93 of PrP^C^ [44]. Whether glypican-1 is this intermediate HSPG awaits further study.

Like heparan sulfate, many of the anti-prion compounds identified to date are, or have the capacity to stack into, large polyanionic chains [45]. This suggests a common mechanism of inhibition of PrP^Sc^ accumulation by these compounds, perhaps by binding to the same or overlapping sites on PrP. In support of this, sulfated glycans have been shown to compete with both Congo red and phosphorothioated oligonucleotides for binding to PrP^C^ [46],[47]. It is possible that some of the anti-prion compounds identified to date exert their effects by antagonising the binding of glypican-1 to PrP^C^ and PrP^Sc^. The ability of exogenous heparin to inhibit the co-immunoprecipitation of glypican-1 with both PrP^C^ and PrP^Sc^ supports such a hypothesis. Thus, such anti-prion compounds may directly disrupt the interaction between PrP^C^/PrP^Sc^ and glypican-1 which is required for optimal prion conversion. Alternatively, the observation that heparin promotes the endocytosis of PrP^C^, may suggest that some anti-prion compounds could act by a similar mechanism, promoting the endocytosis of PrP^C^, thereby directing the protein to late endosomes and/or lysosomes where conversion to PrP^Sc^ is inefficient [15] and/or removing PrP^C^ from the cell surface lipid rafts where glypican-1 facilitates conversion. Following from these results, the design of specific small molecule inhibitors to antagonise the binding of glypican-1 and PrP^C^/PrP^Sc^ may represent a viable therapeutic avenue for the treatment of prion diseases. With any potential treatment for prion diseases that targets PrP^C^, there is the concern that the normal functions of the protein may be adversely affected. In this respect, the lack of effect of glypican-1 depletion on the ability of PrP^C^ to inhibit the cleavage of APP by BACE1 [19] suggests that at least one of the proposed physiological functions of PrP^C^ [48] is independent of its interaction with glypican-1.

Although heparan and other GAGs disrupt prion conversion in both cell and animal models [49]–[54], such compounds stimulate the conversion of PrP^C^ to PrP^Sc^ in cell free systems [38],[55]. From our data this apparent paradox can be explained by exogenous GAGs competitively inhibiting the binding of PrP^C^/PrP^Sc^ to glypican-1 in the cell and animal models, thus not allowing the cellular and infectious forms of PrP to come into close enough contact for conversion to occur (Fig. 9B). While in the cell-free systems the GAGs provide a scaffold to promote the interaction of PrP^Sc^ with PrP^C^ thereby facilitating conversion.

In conclusion, we have identified the cell surface HSPG, glypican-1, as a novel lipid raft targeting determinant for PrP^C^. In addition, we show that depletion of glypican-1 inhibits the formation of PrP^Sc^ in scrapie-infected cells, implying that glypican-1 is a novel cellular cofactor in prion conversion. Furthermore, we show that glypican-1 is not required for one of the proposed physiological functions of PrP^C^, inhibition of the β-secretase cleavage of APP.

## Materials and Methods

### PrP constructs and cell culture

Insertion of the coding sequence of murine PrP containing a 3F4 epitope tag into pIRESneo (Clontech-Takara Bio Europe) and generation of the PrP-TM construct have been reported previously [22]. For stable transfection of the cDNA encoding the PrP constructs, 30 µg DNA was introduced into human SH-SY5Y neuroblastoma cells by electroporation and selection was performed in normal growth medium containing G418 selection antibiotic. Mouse N2a neuroblastoma cells, N2a cells infected with the mouse-adapted 22L scrapie strain (ScN2a) [56] and SH-SY5Y cells were routinely cultured in Dulbecco's modified Eagle's medium supplemented with 10% foetal bovine serum, 50 U/ml penicillin and 0.1 mg/ml streptomycin (all from Invitrogen). Cells were maintained in a humidified incubator at 37°C with 5% CO_2_. For analysis of cell-associated proteins, cells were washed with phosphate-buffered saline (20 mM Na_2_HPO_4_, 2 mM NaH_2_PO_4_, 0.15 M NaCl, pH 7.4) and scraped from the flasks into phosphate-buffered saline. Cells were pelleted by centrifugation at 500 *g* for 5 min. Unless indicated otherwise, pelleted cells were lysed in ice cold lysis buffer (150 mM NaCl, 0.5% (v/v) Triton X-100, 0.5% (w/v) sodium deoxycholate, 50 mM Tris-HCl, pH 7.5) supplemented with a complete protease inhibitor cocktail (Roche Applied Science, Burgess Hill, U.K).

### Endocytosis assay

Cells at confluency were incubated for 1 h at 4°C with 0.5 mg/ml Biotin sulfo-NHS (Sigma-Aldrich, Poole, U.K.). Cells were then incubated for 30 min at 37°C in OptiMEM. Prior to cell lysis, PrP remaining on the cell surface was removed by digestion with trypsin as described previously [23],[24].

### DRM isolation

Cells at confluence were surface biotinylated and then treated as described in individual experiments in the presence of Tyrphostin A23 to prevent PrP^C^ endocytosis [24]. Media was then removed and cells rinsed in phosphate-buffered saline. Cells were subsequently harvested and then resuspended in Mes-buffered saline (25 mM Mes, 150 mM NaCl, pH 6.5) containing 1% (v/v) Triton X-100. Cells were then homogenised by passing through a Luer 21-gauge needle. After centrifugation at 500 *g* for 5 min to pellet cell debris, the supernatant was harvested and made 40% (v/v) with respect to sucrose by addition of an equal volume of 80% (v/v) sucrose. A 1 ml aliquot of the sample was then placed beneath a discontinuous sucrose gradient comprising 3 ml of 30% sucrose and 1 ml of 5% sucrose, both in Mes-buffered saline. The samples were centrifuged at 140,000 *g* in an SW-55 rotor (Beckman Coulter Inc., CA, U.S.A.) for 18 h at 4°C. The sucrose gradients were harvested in 0.5 ml fractions from the base of the gradient and the distribution of proteins monitored by western blot analysis of the individual fractions. Where indicated, biotin-labelled PrP was detected by subsequent immunoprecipitation of epitope-tagged PrP from the individual fractions using antibody 3F4 (Eurogentec Ltd., Southampton, U.K.) and subsequent immunoblotting using horseradish peroxidase-conjugated streptavidin (Thermo Fisher Scientific, Cramlington, U.K.).

### Protein assay and enzyme treatments

Protein was quantified using bicinchoninic acid [57] in a microtitre plate-based assay using bovine serum albumin as standard. To detect PK-resistant PrP^Sc^, cell lysate samples containing 200 µg of protein in a total volume of 200 µl were incubated for 30 min at 37°C with 4 µg PK. The reaction was terminated by the addition of phenylmethanesulfonyl fluoride to a final concentration of 3 mM. Protein in the samples was precipitated by the addition of 800 µl of ice cold methanol and incubated overnight at 4°C. Precipitated protein was pelleted by centrifugation and then resuspended in dissociation buffer (125 mM Tris-HCl pH 8.0, 2% (w/v) sodium dodecyl sulfate, 20% (v/v) glycerol, 100 mM dithiothreitol, bromophenol blue, pH 6.8) prior to SDS-PAGE. To remove heparan sulfate sidechains from HSPGs prior to detection of glypican-1 and syndecan-1 core proteins by immunoblotting, cell lysates (50 µg protein) were incubated for 5 h at 37°C in the presence of 1 m unit heparinase I and 1 m unit heparinase III (both Sigma). To remove cell surface GPI-anchored proteins, cell monolayers were incubated for 1 h at 4°C with 1 U/ml *Bacillus thuringiensis* phosphatidylinositol-specific phospholipase C (PI-PLC) (Sigma-Aldrich).

### SDS-PAGE and western blot analysis

Samples were prepared in dissociation buffer and boiled for 5 min. Proteins were resolved by SDS-PAGE using either 7–17% polyacrylamide gradient gels or 14.5% polyacrylamide gels. For western blot analysis, resolved proteins were transferred to Immobilon P polyvinylidene difluoride membrane (Amersham, Little Chalfont, U.K.). The membrane was blocked by incubation for 1 h with phosphate-buffered saline containing 0.1% (v/v) Tween-20 and 5% (w/v) dried milk powder. Antibody incubations were performed in phosphate-buffered saline-Tween containing 2% (v/v) bovine serum albumin. Antibody 3F4 recognises the 3F4 epitope tag (corresponding to amino acid residues 109–112 of human PrP) at residues 108–111 of the chimeric murine PrP and was used at a dilution of 1∶4000; antibody 6D11 (Eurogentec Ltd.) recognises an epitope within amino acids 93–109 of PrP and was used at 1∶10000; antibody 22C11 (Millipore (UK) Ltd, Livingston, U.K.) recognises amino acid residues 66–81 at the N-terminus of APP and was used at 1∶2500; antibody 1A9 (GlaxoSmithKline, Harlow, U.K.) recognises a neoepitope on the soluble ectodomain fragment of APP derived from β-secretase cleavage (sAPPβ) and was used at 1∶2500; antibody S1 against human glypican-1 was a kind gift from Professor G. David (Flanders Institute for Biotechnology, Belgium) and was used at 1∶2000; polyclonal anti-glypican-1 and anti-syndecan-1 (both Abcam plc, Cambridge, U.K.) were used at 1∶1000; and anti-actin antibody (Sigma) at 1∶5000. Horseradish peroxidase-conjugated streptavidin was used at 1∶2000. Bound antibody was detected using peroxidase-conjugated secondary antibodies in conjunction with the enhanced chemiluminescence detection method (Amersham Biosciences, Amersham, U.K.).

### RNA interference studies

SH-SY5Y cells expressing PrP^C^ were seeded into T80 flasks at 70% confluency and incubated with 500 pmol of a 2 µM Smartpool siRNA solution against glypican-1 or a control Smartpool reagent (Dharmacon Inc., Chicago, U.S.A.) complexed with DharmaFECT-1 transfection regent (Dharmacon Inc.) in serum-free medium. After 4 h, serum was added to 10% (v/v). Cells were then incubated for a further 44 h prior to experimentation. To analyse the role of glypican-1 in the inhibitory action of PrP^C^ on the BACE1 cleavage of APP, SH-SY5Y cells expressing APP695 were transfected with the cDNA encoding PrP^C^ or an empty pIRES*neo* vector and treated with glypican-1 or control Smartpool reagents. After 30 h the medium was changed to OptiMEM (Invitrogen), and the cells incubated for a further 24 h. Cells were pelleted and the medium harvested and centrifuged at 1000 *g* for 5 min to remove residual cell debris. Medium was then concentrated 50-fold using Vivaspin centrifugal concentrators (10 000 MW cut off). To assess the role of glypcian-1 on prion conversion ScN2a cells were treated with 500 pmol of the following 2 µM siRNA solutions: Smartpool glypican-1 solution; one of four individual duplexes targeted to glypican-1; Smartpool syndecan-1 solution or control Smartpool siRNA reagent. Cells were treated twice over a 96 h period with siRNA (at 0 and 48 h). After 96 h cells were harvested and lysed.

### Co-immunoprecipitation

Co-immunoprecipitation of PrP isoforms and glypican-1 were performed as described previously [58]. Briefly, SH-SY5Y cells expressing PrP^C^, N2a cells or ScN2a cells were grown to confluence and then harvested. Cells were lysed using chilled immunoprecipitation lysis buffer (150 mM NaCl, 10 mM EDTA, 10 mM KH_2_PO_4_, pH 7.5) containing, where indicated 2% (v/v) Triton X-100, 1% *n*-octyl-β-D-glucopyranoside (octyl glucoside) or 1% N-laurylsarcosine (sarkosyl). Cell debris were removed by centrifugation and then lysates precleared for 30 min with 0.5% (w/v) protein A-Sepharose. The protein A-Sepharose was removed by centrifugation and the supernatant incubated overnight at 4°C with anti-PrP antibody (6H4) or anti-glypican-1 antibody. Protein A-Sepharose was added to 0.5% (w/v) to the samples and incubation proceeded at 37°C for 1 h. Immunocomplexes were pelleted and the pellet washed six times with 150 mM NaCl, 10 mM Tris-HCl, pH 7.4 containing 0.2% (v/v) Tween-20.

### Immunofluorescence microscopy

Cells were seeded onto coverslips and grown to 50% confluency. Cells were then fixed with 4% (v/v) paraformaldehyde/0.1% (v/v) glutaraldehyde in PBS for 15 min, and then blocked for 1 h in PBS containing 5% (v/v) fish skin gelatin (Sigma-Aldrich). Coverslips were then incubated with anti-PrP antibody 3F4 and anti-glypican-1 antibody. Finally, coverslips were incubated with the appropriate fluorescent probe-conjugated secondary antibodies (Molecular Probes, Eugene, U.S.A.) for 1 h and mounted on slides using fluoromount G mounting medium (SouthernBiotech, Alabama, U.S.A). Cells were visualised using a DeltaVision Optical Restoration Microscopy System (Applied Precision Inc., Washington, USA). Data were collected from 30–40 0.1 µm thick optical sections, and 3-D datasets were deconvolved using the softWoRx programme (Applied Precision Inc.). The presented images represent individual Z-slices taken from the middle of the cell.

### Assessment of cell number by Hoescht 33342 staining

ScN2a cells (1×10^4^ per well) in 96-well tissue culture plates were cultured overnight in complete medium. After 24 h, cells were treated with the indicated siRNA duplexes. Cells were fixed in 70% ethanol at room temperature for 5 min, and the adherent cell monolayers were stained with the DNA-binding fluorochrome Hoescht 33342 (8.8 µM). Once dry, the fluorescence of each well was measured on a Synergy HT (Bio-Tek) fluorescent plate reader (350 nm excitation and 450 nm emission wavelengths) in order to determine the cell number in each well.

### Statistical analysis

Data are expressed as means (± s.e.m.). Experiments were performed in triplicate and repeated on at least three occasions. Statistical analysis was performed using student's t-test. P-values <0.05 were taken as statistically significant.
